# Lower Frequency of HLA-DRB1 Type 1 Diabetes Risk Alleles in Pediatric Patients with MODY

**DOI:** 10.1371/journal.pone.0169389

**Published:** 2017-01-04

**Authors:** Inés Urrutia, Rosa Martínez, Tamara López-Euba, Teresa Velayos, Idoia Martínez de LaPiscina, José Ramón Bilbao, Itxaso Rica, Luis Castaño, on behalf of The Spanish Group for the Study of MODY and Type 1 diabetes

**Affiliations:** BioCruces Health Research Institute, Cruces University Hospital, UPV-EHU, CIBERDEM, CIBERER, Barakaldo, Spain; Istanbul University, TURKEY

## Abstract

**Objective:**

The aim of this study was to determine the frequency of susceptible HLA-DRB1 alleles for type 1 diabetes in a cohort of pediatric patients with a confirmed genetic diagnosis of MODY.

**Materials and Methods:**

160 families with a proband diagnosed with type 1 diabetes and 74 families with a molecular diagnosis of MODY (61 *GCK*-MODY and 13 *HNF1A*-MODY) were categorized at high definition for HLA-DRB1 locus. According to the presence or absence of the susceptible HLA-DRB1 alleles for type 1 diabetes, we considered three different HLA-DRB1 genotypes: 0 risk alleles (no DR3 no DR4); 1 risk allele (DR3 or DR4); 2 risk alleles (DR3 and/or DR4).

**Results:**

Compared with type 1 diabetes, patients with MODY carried higher frequency of 0 risk alleles, OR 22.7 (95% CI: 10.7–48.6) and lower frequency of 1 or 2 risk alleles, OR 0.53 (95% CI: 0.29–0.96) and OR 0.06 (95% CI: 0.02–0.18), respectively.

**Conclusions:**

The frequency of HLA-DRB1 risk alleles for type 1 diabetes is significantly lower in patients with MODY. In children and adolescents with diabetes, the presence of 2 risk alleles (DR3 and/or DR4) reduces the probability of MODY diagnosis, whereas the lack of risk alleles increases it. Therefore, we might consider that HLA-DRB1 provides additional information for the selection of patients with high probability of monogenic diabetes.

## Introduction

Diabetes mellitus represents a group of metabolic disorders characterized by increased levels of blood glucose resulting from defects in insulin secretion, insulin action, or both.

Type 1 diabetes mellitus develops primarily in youth as a result of autoimmune destruction of the pancreatic beta-cells and is characterized by absolute insulin deficiency. In this disorder, glutamic acid decarboxylase (GADA), tyrosine phosphatase (IA2A), zinc transporter 8 (ZnT8A) and insulin autoantibodies (IAA) are currently recognized as autoimmune process markers [1, 2]. The pathogenesis of the disease is determined by complex interactions between several genetic loci and environmental factors. The first genetic contribution strongly associated with type 1 diabetes was found within the HLA region on chromosome 6p21. Susceptibility to, and protection against development of autoimmune diabetes are associated with the highly polymorphic sequences of the HLA class II genes. The strongest susceptibility haplotypes described are HLA DRB1*03-DQA1*0501-DQB1*0201 and HLA DRB1*04-DQA1*0301-DQB1*0302 especially when both are present in the genotype. [3]

Maturity-onset diabetes of the young (MODY) refers to the most common form of monogenic diabetes characterized by early onset (usually before age 25), non-autoimmune diabetes with autosomal dominant inheritance and additional features unique to different MODY subtypes [4]. Although more than 10 different genes have been associated with MODY, mutations in the *GCK* and *HNF1A* genes are the most frequent causes of MODY in all populations studied, including the Spanish population. They account for approximately 80% of the cases [5, 6].

MODY can be undiagnosed or misdiagnosed as type 1 or type 2 diabetes [7]. Genetic testing to confirm the clinical diagnosis of MODY has important implications in patient management such as changes in treatment, improvement in the glycemic control avoiding complications and identification of asymptomatic family members [8].

As molecular genetic testing is expensive and arduous it should be limited to those cases more likely to be MODY [9]. In the last few years several analytical tests have been proposed in order to improve the selection of patients to whom the genetic study should be applied [10–14]. However, none of the markers described so far are able to differentiate definitely between these 2 distinct types of diabetes [15, 16].

The aim of this study was to identify susceptible HLA-DRB1 alleles for type 1 diabetes in a Spanish youth population and to determine their frequency in a cohort of pediatric patients with a confirmed genetic diagnosis of MODY.

## Materials and Methods

### Patients

This study included two different groups of Spanish pediatric patients with diabetes. The first group consisted of 160 unrelated families, comprising a new onset patient with type 1 diabetes and their parents. Diagnosis of the disease was done according to the World Health Organization (WHO) criteria [17]. All patients with type 1 diabetes (average onset age 8.8 ± 3.9 years) were positive for one or more of the measured pancreatic autoantibodies (IAA, GADA and IA2A) determined in serum at diagnosis, using previously described standardized radio-assays [1].

The second group consisted of 74 unrelated families, comprising a new onset patient with MODY (61 *GCK*-MODY and 13 *HNF1A*-MODY) and their parents. All *GCK*-MODY (average onset age 9.2 ± 5.4 years) and *HNF1A*-MODY (average onset age 14.2 ± 3.8 years) new onset cases had negative pancreatic autoantibodies. Characteristics of the study population are shown in Table 1.

**Table 1:** Characteristics of the population included in the study stratified by HLA-DRB1 genotype. Data are shown as % or mean ± SD; T1D, type 1 diabetes; n/a, not applicable; DRX, corresponds to any HLA-DRB1 allele different from DR3 and DR4; DRB1*0403 allele is considered DRX.

		T1D (n = 160)	MODY (n = 74)	Controls (n = 75)
Females		53.7	56.7	49.3
Age at diagnosis (years)		8.8 ± 3.9	10.0 ± 5.9	n/a
2 risk alleles	DR3/DR4	27.5	2.7	1.3
	DR3/DR3	13.7	0.0	0.0
	DR4/DR4	6.9	2.7	0.0
1 risk allele	DR3/DRX	25.6	16.2	18.7
	DR4/DRX	18.7	13.5	17.3
0 risk alleles	DRX/DRX	7.5	64.9	62.7

The study was approved by the Clinical Research Ethics Committee (CEIC) from Cruces University Hospital, and written informed consent was obtained from all subjects or their parents.

### Genotyping Methods

DNA extraction was performed with QiAamp DNA blood kit (Qiagen, Hilden, NRW, Germany) according to manufacturer's protocol.

#### HLA-DRB1 typing

Polymerase chain reaction sequence-specific oligonucleotide method (PCR-SSO) combined with Luminex technology was carried out using LABType RSSOH2B1 (HLA-DRB1-HD) commercial kit (One Lambda, Inc., Canoga Park, CA, USA). The protocol comprised the DNA amplification process using a group specific primer, hybridization with sequence-specific oligonucleotide probes (SSO), reading on a special device (LABScan™100) and software interpretation (HLA Fusion™). All procedures were performed according to manufacturers' instructions.

#### Mutation screening

Genetic testing for mutations in *GCK* and *HNF1A* genes was performed by bidirectional Sanger sequencing (ABI 3130 xl Genetic Analyser, Applied Biosystems) as described [6].

### Statistical Analysis

To identify susceptible HLA-DRB1 alleles for type 1 diabetes in our population, an AFBAC study was performed. From the four parental HLA-DRB1 alleles in each family, those never transmitted to a member with type 1 diabetes were categorized as “non-diabetic alleles”, and those carried by patients with type 1 diabetes were classified as “diabetic alleles”.

To compare the distribution of HLA-DRB1 genotypes between MODY and type 1 diabetes, a case-control study was performed considering the new onset patients with MODY and with type 1 diabetes.

To compare the distribution of HLA-DRB1 genotypes between MODY patients and control subjects, a case-control study was performed. In this case, *GCK*-MODY or *HNF1A*-MODY mutation carriers were compared with healthy family members who did not carry the mutation (healthy controls), ensuring that there was no filial relationship (wives and husbands) among these individuals.

Frequencies were compared using Pearson´s chi-square analysis and Fisher exact test when necessary. The significance level was defined as *p*<0.05. We evaluated the results using SPSS software (version 21; SPSS Inc., Chicago, IL).

## Results

**Fig 1:**
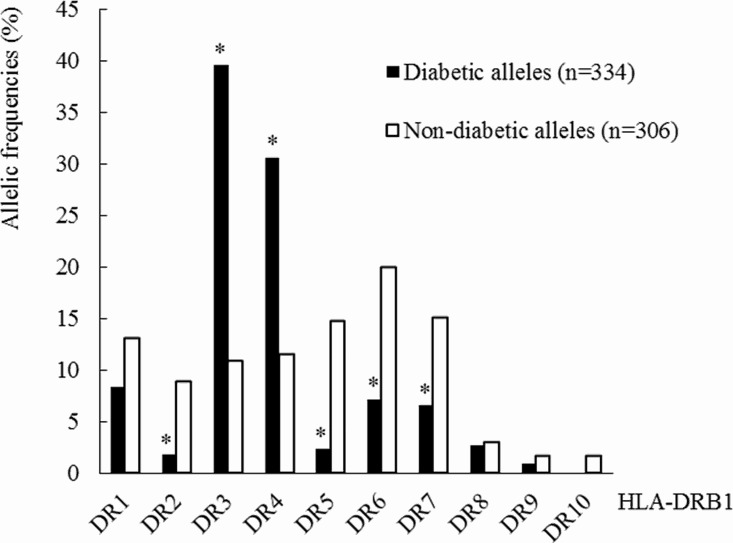
Distribution of the frequencies of diabetic and non-diabetic HLA-DRB1 alleles in the Spanish population. DR2 (DRB1*15, DRB1*16); DR5 (DRB1*11, DRB1*12); DR6 (DRB1*13, DRB1*14). Asterisks indicate cases in which differences in the allelic frequency are significant *p<0.001.

The distribution of the frequencies of diabetic and non-diabetic HLA-DRB1 alleles in Spanish youth population is shown in Fig 1. Our dataset showed an increase of DR3 and DR4 alleles in the diabetic alleles group compared with the non-diabetic ones, OR 5.41 (95% CI: 3.48–8.44) and OR 3.40 (95% CI: 2.19–5.31), respectively. On the contrary, DR2, DR5, DR6 and DR7 allelic frequencies were higher in the non-diabetic alleles group, OR 0.19 (95% CI: 0.06–0.48); 0.14 (0.06–0.32); 0.31 (0.18–0.53); 0.40 (0.23–0.70), respectively.

Among the most prevalent HLA-DRB1*04 subtypes, *0401, *0402, *0404 and *0405 were more represented in the diabetic alleles group. Conversely, subtype *0403 was more represented in the non-diabetic alleles group (12/35, 34.3%) than in the diabetic alleles group (3/102, 2.9%) p<0.001. Due to the fact that *0403 did not confer susceptibility to type 1 diabetes, OR 0.06 (95% CI: 0.01–0.25), the *0403 allele is excluded from the DR4 alleles group in the subsequent analysis.

**Fig 2:**
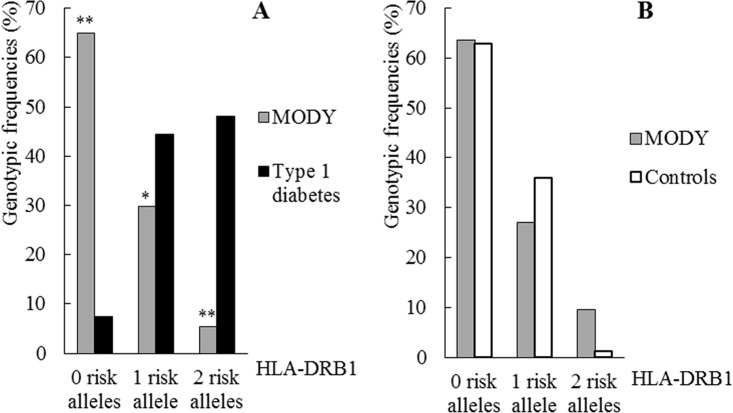
Distribution of HLA-DRB1 genotypes in patients with type 1 diabetes, patients with MODY and control subjects. 0 risk alleles (no DR3 no DR4); 1 risk allele (DR3 or DR4); 2 risk alleles (DR3/3, DR4/4 or DR3/4). (A) Two groups are compared: probands with MODY (n = 74) and probands with type 1 diabetes (n = 160). HLA-DRB1 genotype frequencies differed significantly between groups (*p*<0.001). Comparisons of each of the genotypes separately showed significant differences in all cases **p<0.001, *p<0.03. (B) Two groups are compared: patients with MODY (n = 63) and healthy controls (n = 75). There were no significant differences between groups (p = 0.067).

HLA-DRB1 genotype frequency distribution in patients with type 1 diabetes, patients with MODY and healthy controls is shown in Fig 2. Depending on the presence or absence of the susceptible alleles for type 1 diabetes, DR3 and DR4, we have grouped the genotypes in 3 categories: 0 risk alleles (no DR3 no DR4 present); 1 risk allele (only one allele DR3 or DR4 is present); 2 risk alleles (individuals homozygous for DR3 or DR4, or DR3-DR4 heterozygous). As shown in Fig 2A, the distribution of frequencies of the HLA-DRB1 genotypes was significantly different in MODY compared to patients with type 1 diabetes (*p*<0.001). In this case, the comparison of each of the HLA-DRB1 genotypes *versus* the remaining categories, showed that the frequency of genotypes with two risk alleles was significantly lower in MODY than in patients with autoimmune diabetes, OR 0.06 (95% CI: 0.02–0.18). Furthermore, the frequency of genotypes carrying a single risk allele was also lower in MODY patients, OR 0.53 (95% CI: 0.29–0.96). A remarkable higher proportion of MODY patients carried genotypes without risk alleles when compared with patients with type 1 diabetes, OR 22.77 (95% CI: 10.7–48.6). There were no significant differences in the HLA-DRB1 genotype frequency distribution between MODY patients and healthy controls (*p* = 0.067) Fig 2B.

To examine the validity of HLA-DRB1 genotyping in the selection of pediatric patients for genetic study of MODY, we categorized the groups as associated with MODY, when one or no risk alleles were present, and as associated with type 1 diabetes, when two risk alleles were present. This test showed a sensitivity of 94.6% with a specificity of 48.1% when discerning MODY from type 1 diabetes. The positive predictive value for the identification of MODY was 45.8% for one or no risk allele and the negative predictive value was 95.1% for two risk alleles. The test accuracy to detect monogenic diabetes was 62.8%

## Discussion

Our data support the association of DR3 and DR4 alleles with type 1 diabetes and also the protective role of the DRB1*0403 allele, as already described in Caucasian populations [3, 18, 19]. Furthermore, the HLA-DRB1 genotype distribution is similar between MODY and control subjects. This fact provides evidence that, unlike type 1 diabetes, the development of MODY is not associated with HLA-DRB1 genotype.

The difference in HLA-DRB1 genotype distribution between MODY and type 1 diabetes is especially remarkable when patients carry two risk alleles (5.4% MODY *vs*. 48.1% type 1 diabetes) or none (64.9% MODY *vs*. 7.5% type 1 diabetes). When only one risk allele is present, the difference has less importance (29.7% MODY *vs*. 44.3% type 1 diabetes) so the presence of a single risk allele is not conclusive to differentiate between autoimmune diabetes and MODY and for this reason HLA-DRB1 genotyping has the low specificity shown before (48.1%). Other clinical and/or analytical data should be considered to achieve a better specificity.

These results suggest it might be interesting to perform HLA-DRB1 test before initiating the genetic study of MODY: as two risk alleles are present in just 5% of patients with monogenic diabetes, in this case genetic testing for MODY should be performed only if other clinical and/or analytical data suggest it. In the same way, the lack of risk alleles is found in just 7.5% of patients with type 1 diabetes. In this case, the possibility of proposing an unnecessary genetic study is low and we would recommend doing it. For the accurate interpretation of the test, it is important to note that the low prevalence of MODY in children with diabetes, 1–3% [20], shifts the diagnosis towards type 1 diabetes so that the presence of 2 risk alleles effectively rules out MODY. For the same reason, the probability to mistakenly select a patient for a genetic study increases. Nevertheless, to minimize this, it is of utmost importance to take into account all the available clinical and analytical data to support the decision to include a patient in a genetic study to confirm MODY.

Additionally, HLA-DRB1 typing might be useful in particular situations when clinical and/or analytical data are not conclusive. This can be the case when the family history of diabetes is not available or a patient carries a *de novo* mutation. It has been recently published that the prevalence of *de novo* mutations in MODY may be more frequent than previously assumed [21], and in our data set it is 6.7%. This situation could discourage medical doctors to perform genetic testing and therefore MODY could be misdiagnosed. Another particular situation includes unavailability of serum at the onset of the disease and/or negative pancreatic autoantibodies. Pancreatic autoantibody screening at the onset of the disease is, so far, the most specific test described for autoimmune diabetes differentiation. Although 10% of patients with type 1 diabetes may present negative pancreatic autoantibodies [5], we recommend this test as the first step for the differentiation of MODY. This analysis has to be made close to the moment of diagnosis because autoantibody titers decrease as a function of longer duration type 1 diabetes [22]. Inability to detect pancreatic autoantibodies could reject a type 1 diabetes diagnosis and could suggest the possibility of being MODY. If antibodies are positive and the patient has evident clinical features of autoimmune diabetes, the diagnosis would be type 1 diabetes and then, HLA-DRB1 genotyping could just confirm it.

A two-SNPs screening test to identify the highest risk HLA genotype for patients with type 1 diabetes has been published [23]. This method could be used to replace HLA-DRB1 typing even though it cannot identify the DR3 and DR4 homozygous genotypes that also confer a remarkable risk to type 1 diabetes and it may not be accessible everywhere. On the contrary, HLA typing is a well standardized technique in clinical practice, is available in most hospitals in Spain and is able to identify all HLA-DRB1 genotypes associated with type 1 diabetes.

There are several limitations of this study. It is well known that MODY can be misdiagnosed not only as type 1 but also as type 2 diabetes. Moreover, it is unlikely that HLA-DRB1 could play any role in discriminating MODY from type 2 diabetes. Thus, we could consider a limitation in our study not having included a group of patients with type 2 diabetes in the analysis. However, the current prevalence of type 2 diabetes in our Caucasian youth population is very low [24, 25]. Therefore, it is unlikely that MODY may be misdiagnosed as type 2 diabetes in this pediatric population. We should also point out that our study has been exclusively performed in Caucasian youth population, thus, results should be interpreted with caution when considering other ethnic groups or adult populations where type 2 diabetes is more prevalent. Finally, the type 1 diabetes group includes exclusively pediatric patients with positive pancreatic autoantibodies and we did not consider the C-peptide value.

In summary, the frequency of HLA-DRB1 risk alleles for type 1 diabetes is significantly lower in patients with MODY. In children and adolescents with diabetes, the presence of 2 risk alleles (DR3 and/or DR4) reduces the probability of MODY diagnosis, whereas the lack of risk alleles increases it. HLA-DRB1 genotyping together with all known biomarkers, could be helpful in order to select pediatric patients with high probability of monogenic diabetes.

## Spanish Group for the Study of MODY and Type 1 Diabetes

H. Basurto—Bizkaia (Concepción Fernández-Ramos, Javier Núñez), H. Costa del Sol—Málaga (Leopoldo Tapia), H. Cruces—Bizkaia (Anibal Aguayo, Sonia Gaztambide, Teba González-Frutos, Gema Grau, Amaia Rodríguez-Estévez, Amaia Vela), H. Marqués de Valdecilla—Santander (Cristina Luzuriaga), H. Miguel Servet—Zaragoza (Gracia M Lou), Complejo Hospitalario de Navarra (María Chueca), H. Ramón y Cajal—Madrid (Raquel Barrio), H. Reina Sofía (Joaquín Gómez-Vázquez), H. San Pedro de Alcántara—Cáceres (Jesús González de Buitrago), H. Severo Ochoa—Madrid (Beatriz García-Cuartero), H. Son Espases—Palma de Mallorca (Diego de Sotto), H. Vall d´Hebron—Barcelona (Ariadna Campos), H. Virgen del Rocío—Sevilla (Ana L Gómez-Gila)
